# PIRK: Scalable Interval Reachability Analysis for High-Dimensional Nonlinear Systems

**DOI:** 10.1007/978-3-030-53288-8_27

**Published:** 2020-06-13

**Authors:** Alex Devonport, Mahmoud Khaled, Murat Arcak, Majid Zamani

**Affiliations:** 8 Microsoft Research Lab, Redmond, WA USA; 9 University of Southern California, Los Angeles, CA USA; 10 University of California, Berkeley, Berkeley, CA USA; 11 Technical University of Munich, Munich, Germany; 12 University of Colorado, Boulder, Boulder, CO USA; 13 Ludwig Maximilian University, Munich, Germany

**Keywords:** Reachability analysis, ODE integration, Runge-Kutta method, Mixed monotonicity, Monte Carlo simulation, Parallel algorithms

## Abstract

Reachability analysis is a critical tool for the formal verification of dynamical systems and the synthesis of controllers for them. Due to their computational complexity, many reachability analysis methods are restricted to systems with relatively small dimensions. One significant reason for such limitation is that those approaches, and their implementations, are not designed to leverage parallelism. They use algorithms that are designed to run serially within one compute unit and they can not utilize widely-available high-performance computing (HPC) platforms such as many-core CPUs, GPUs and Cloud-computing services.

This paper presents PIRK, a tool to efficiently compute reachable sets for general nonlinear systems of extremely high dimensions. PIRK can utilize HPC platforms for computing reachable sets for general high-dimensional non-linear systems. PIRK has been tested on several systems, with state dimensions up to 4 billion. The scalability of PIRK’s parallel implementations is found to be highly favorable.

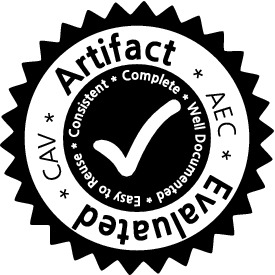



## Introduction

Applications of safety-critical cyber-physical systems (CPS) are growing due to emerging IoT technologies and the increasing availability of efficient computing devices. These include smart buildings, traffic networks, autonomous vehicles, truck platooning, and drone swarms, which require reliable bug-free software that perform in real-time and fulfill design requirements. Traditional simulation/testing-based strategies may only find a small percentage of the software defects and the repairs become much costly as the system complexity grows. Hence, in-development verification strategies are favorable since they reveal the faults in earlier stages, and guarantee that the designs satisfy the specifications as they evolve through the development cycle. Formal methods offer an attractive alternative to testing- and simulation-based approaches, as they can verify whether the specifications for a CPS are satisfied for all possible behaviors from a set of the initial states of the system. *Reachable sets* characterize the states a system can reach in a given time range, starting from a certain initial set and subjected to certain inputs. They play an important role in several formal methods-based approaches to the verification and controller synthesis. An example of this is *abstraction-based* synthesis [1–4], in which reachable sets are used to construct a finite-state “abstraction” which is then used for formal synthesis.

Computing an exact reachable set is generally not possible. Most practical methods resort to computing over-approximations or under-approximations of the reachable set, depending on the desired guarantee. Computing these approximations to a high degree of accuracy is still a computationally intensive task, particularly for high-dimensional systems. Many software tools have been created to address the various challenges of approximating reachable sets. Each of these tools uses different methods and leverages different system assumptions to achieve different goals related to computing reachable sets. For example, CORA [5] and SpaceEx [6] tools are designed to compute reachable sets of high accuracy for very general classes of nonlinear systems, including hybrid ones. Some reachability analysis methods rely on specific features of dynamical systems, such as linearity of the dynamics or sparsity in the interconnection structure [7–9]. This allows computing the reachable sets in shorter time or for relatively high-dimensional systems. However, it limits the approach to smaller classes of applications, less practical specifications, or requires the use of less accurate (e.g., linearized) models.

Other methods attack the computational complexity problem by computing reachable set approximations from a limited class of set representations. An example of limiting the set of allowed overapproximations are *interval reachability* methods, in which reachable sets are approximated by Cartesian products of intervals. Interval reachability methods allow for computing the reachable sets of very general non-linear and high-dimensional systems in a short amount of time. They also pose mild constraints on the systems under consideration, usually only requiring some kind of boundedness constraint instead of a specific form for the system dynamics. Many reachability tools that are designed to scale well with state dimension focus on interval reachability methods: these include Flow$^*$ [10], CAPD [11], C2E2 [12], VNODE-LP [13], DynIbex [14], and TIRA [15].

Another avenue by which reachable set computation time can be reduced, which we believe has not been sufficiently explored, is the use of parallel computing. Although most reachability methods are presented as serial algorithms, many of them have some inherent parallelism that can be exploited. One example of a tool that exploits parallelism is XSpeed [16], which implements a parallelized version of a support function-based reachability method. However, this parallel method is limited to linear systems, and in some cases only linear systems with invertible dynamics. Further, the parallelization is not suitable for massively parallel hardware: only some of the work (sampling of the support functions) is offloaded to the parallel device, so only a relatively small number of parallel processing elements may be employed.

In this paper, we investigate the parallelism for three interval reachability analysis methods and introduce PIRK, the Parallel Interval Reachability Kernel. PIRK uses *simulation-based* reachability methods [17–19], which compute rigorous approximations to reachable sets by integrating one or more systems of ODEs. PIRK is developed in C++ and OpenCL as an open-source^1^ *kernel* for pFaces [20], a recently introduced acceleration ecosystem. This allows PIRK to be run on a wide range of computing platforms, including CPUs clusters, GPUs, and hardware accelerators from any vendor, as well as cloud-based services like AWS.

The user looking to use a reachability analysis tool for formal verification may choose from an abundance of options, as our brief review has shown. What PIRK offers in this choice is a tool that allows for massively parallel reachability analysis of high-dimensional systems with an application programming interface (API) to easily interface with other tools. To the best of our knowledge, PIRK is the first and the only tool that can compute reachable sets of general non-linear systems with dimensions beyond the billion. As we show later in Sect. 5, PIRK computes the reachable set for a traffic network example with 4 billion dimension in only 44.7 min using a 96-core CPU in Amazon AWS Cloud.

**Fig. 1.**
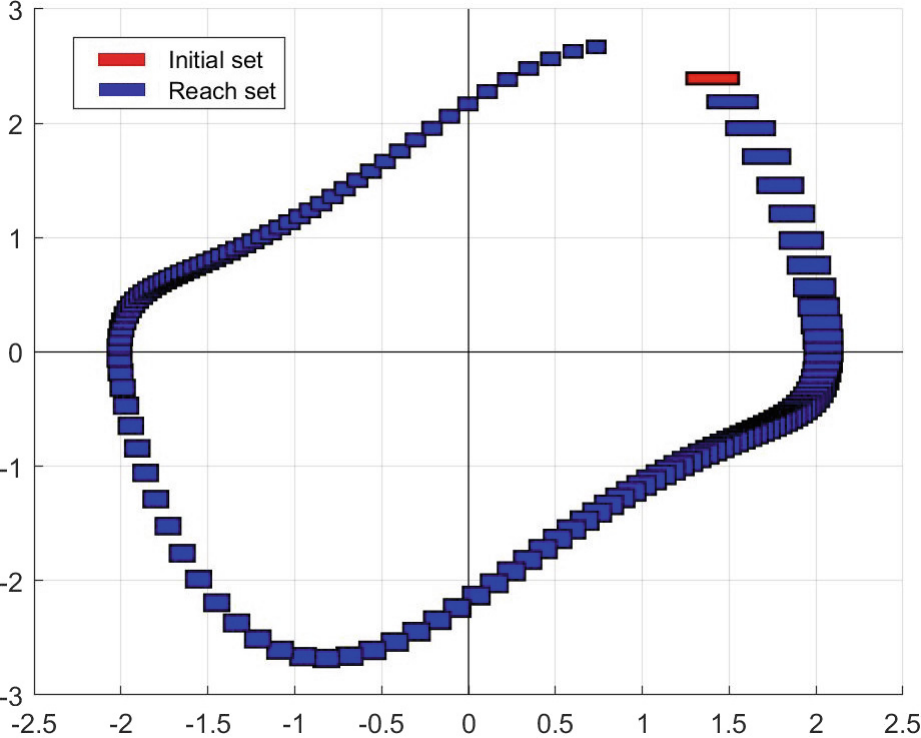
An example of an Interval Reachability problem for a nonlinear system. Red rectangle: initial set. Blue rectangles: reachable sets for several final times $t_1$. (Color figure online)

## Interval Reachability Analysis

Consider a nonlinear system with dynamics $\dot{x} = f(t,x,p)$ with state $x\in \mathbb {R}^{n}$, a set of initial states $\mathcal {X}_0$, a time interval $[t_0,t_1]$, and a set of time-varying inputs $\mathcal {P}$ defined over $[t_0,t_1]$. Let $\varPhi (t;t_0,x_0,p)$ denote the state of the system, at time *t*, of the trajectory beginning at time $t_0$ at initial state $x_0$ under input *p*. We assume the systems are continuous-time.

The finite-time forward reachable set is defined as

$$\begin{aligned} R_{t_0,t_1} = \{\varPhi (t_1;t_0,x,p) | x\in \mathcal {X}_0,p\in \mathcal {P}\}. \end{aligned}$$

For the problem of *interval* reachability analysis, there are a few more constraints on the problem structure. An *interval set* is a set of the form $[\underline{a},\overline{a}]=\{a: \underline{a} \le a \le \overline{a} \}$, where $\le$ denotes the usual partial order on real vectors, that is the partial order with respect to the positive orthant cone. The vectors $\underline{a}$ and $\overline{a}$ are the lower and upper bounds respectively of the interval set. An interval set can alternatively be described by its center $a^*=\frac{1}{2}(\overline{a}+\underline{a})$ and half-width $[a]=\frac{1}{2}(\overline{a}-\underline{a})$. In interval reachability analysis, the initial set must be an interval, and inputs values restricted to an interval set, i.e. $p(t)\in [\underline{p},\overline{p}]$, and the reachable set approximation must also be an interval (Fig. 1). Furthermore, certain methods for computing interval reachable sets require further restrictions on the system dynamics, such as the state and input Jacobian matrices being bounded or sign-stable.

### Methods to Compute Interval Reachable Sets

PIRK computes interval reachable sets using three different methods, allowing for different levels of tightness and speed, and which allow for different amounts of additional problem data to be used.

The *Contraction/Growth Bound* method [4, 21, 22] computes the reachable set using component-wise contraction properties of the system. This method may be applied to input-affine systems of the form $\dot{x}=f(t,x) + p$. The growth and contraction properties of each component of the system are first characterized by a *contraction matrix* *C*. The contraction matrix is a component-wise generalization of the matrix measure of the Jacobian $J_x=\partial f / \partial x$ [19, 23], satisfying $C_{ii} \ge J_{x,ii}(t,x)$ for diagonal Jacobian elements $J_{x,ii}(t,x)$, and $C_{ij} \ge |J_{x,ij}(t,x)|$ for off-diagonal Jacobian elements $J_{x,ij}(t,x)$. The method constructs a reachable set over-approximation by separately establishing its *center* and *half-width*. The center is found by simulating the trajectory of the center of the initial set, that is as $\varPhi (t_1;t_0,x^*,p^*)$. The half width is found by integrating the *growth dynamics* $\dot{r}= g(r,p) = Cr + [p],$ where $[p]=\frac{1}{2}(\overline{p}-\underline{p})$, over $[t_0,t_1]$ with initial condition $r(t_0)=[x]=\frac{1}{2}(\overline{x}-\underline{x})$.

The *Mixed-Monotonicity* method [24] computes the reachable set by separating the increasing and decreasing portions of the system dynamics in an auxiliary system called the *embedding system* whose state dimension is twice that of the original system [25]. The embedding system is constructed using a *decomposition function* $d(t,x,p,\hat{x},\hat{p})$, which encodes the increasing and decreasing parts of the system dynamics and satisfies $d(t,x,p,x,p)=f(t,x,p)$. The evaluation of a single trajectory of the embedding system can be used to find a reachable set over-approximation for the original system.

The *Monte Carlo* method computes a probabilistic approximation to the reachable set by evaluating the trajectories of a finite number *m* of pairs *sample points* ($x_0^{(i)}$, $p^{(i)}$) in the initial set and input set, and selecting the smallest interval that contains the final points of the trajectories. Unlike the other two methods, the Monte Carlo method is restricted to constant-valued inputs, i.e. inputs of the form $p(t)=p$, where $p\in [\underline{p},\overline{p}]$. Each sampled initial state $x_0^{(i)}$ is integrated over $[t_0,t_1]$ with its input $p^{(i)}$ to yield a final state $x_1^{(i)}$. The interval reachable set is then approximated by the elementwise minimum and maximum of the $x_1^{(i)}$. This approximation satisfies a probabilistic guarantee of correctness, provided that enough sample states are chosen [26]. Let $[\underline{R},\overline{R}]$ be the approximated reachable set, $\epsilon , \delta \in (0,1)$, and $m \ge (\frac{2n}{\epsilon })\log \left( \frac{2n}{\delta } \right) .$ Then, with probability $1-\delta$, the approximation $[\underline{R},\overline{R}]$ satisfies $P(R_{t_0,t_1} \backslash [\underline{R},\overline{R}]) \le \epsilon ,$ where *P*(*A*) denotes the probability that a sampled initial state will yield a final state in the set *A*, and $\backslash$ denotes set difference. The probability that a sampled initial state will be sent to a state outside the estimate (the “accuracy” of the estimate) is quantified by $\epsilon$. Improved accuracy (lower $\epsilon$) increases the sample size as $O(1/\epsilon )$. The probability that running the algorithm will fail to give an estimate satisfying the inequality (The “confidence”) is quantified by $\delta$. Improved confidence (lower $\delta$) increases the sample size by $O(\log (1/\delta ))$.

## Parallelization

The bulk of the computational work in each method is spent in ODE integration. Hence, the most effective approach by which to parallelize the three methods is to design a parallel ODE integration method. There are several available methods for parallelizing the task of ODE integration. Several popular methods for parallel ODE integration are parallel extensions of Runge-Kutta integration methods, which are the most popular serial methods for ODE integration.

PIRK takes advantage of the task-level parallelism in the Runge-Kutta equations by evaluating each state dimension in parallel. This parallelization scheme is called *parallelization across space* [27]. PIRK specifically uses a space-parallel version of the fourth-order Runge-Kutta method, or space-parallel RK4 for brevity. In space-parallel RK4, each parallel thread is assigned a different state variable to evaluate the intermediate update equations. After each intermediate step, the threads must synchronize to construct the updated state in global memory. Space-parallel RK4 can use as many parallel computation elements as there are state variables: since PIRK’s goal is to compute reachable sets for extremely high-dimensional systems, this is sufficient in most cases.

The space-parallel scheme is not hardware-specific, and may be used with any parallel computing platform. PIRK is similarly hardware-agnostic: the pFaces ecosystem, for which PIRK is a kernel, provides a common interface to run on a variety of heterogeneous parallel computing platforms. The only difference between platforms that affects PIRK is the number of available parallel processing elements (PEs).

## Complexity of the Parallelized Methods

The parallelized implementations of the three reachability methods described in Sect. 2.1 use space-parallel RK4 to perform almost all computations other than setting up initial conditions. We can therefore find the time and memory complexity of each method by analyzing the complexity of space-parallel RK4 and counting the number of times each method uses it.

For a system with *n* dimensions, space-parallel RK4 scales linearly as the number of PEs (denoted by *P*) increases. In a computer with a single PE (i.e., $P = 1$), the algorithm reduces to the original serial algorithm. Then, suppose that a parallel computer has $P \le n$ PEs of the same type. We assume a computational model under which instruction overhead and latency from thread synchronization are negligible, memory space has equal access time from all processing elements, and the number of parallel jobs can be evenly distributed among the P processing elements.^2^ Under this *parallel random-access machine* model [28], the time complexity of space-parallel RK4 is reduced by a factor of *P*: each PE is responsible for computing *n*/*P* components of the state vector. Therefore, for fixed initial and final times $t_0$ and $t_1$, the time complexity of the algorithm is $O(\frac{n}{P})$.

The parallel version of the contraction/growth bound method uses space-parallel RK4 twice. First, it is used to compute the solution of the system’s ODE *f* for the center of the initial set $\mathcal {X}_0$. Then, it is used to compute the growth/contraction of the initial set $\mathcal {X}_0$ by solving the ODE *g* of the growth dynamics. Since this method uses a fixed number of calls of space-parallel RK4, its time complexity is also $O(\frac{n}{P})$ for a given $t_0$ and $t_1$.

The parallelized implementation of the mixed-monotonicity method uses space-parallel RK4 only once, in order to integrate the 2*n*-dimensional embedding system. This means that the mixed-monotonicity method also has a time complexity of $O(\frac{n}{P})$ for fixed $t_0$ and $t_1$. However, the mixed-monotonicity method requires twice as much memory as the growth bound method, since it runs space-parallel RK4 on a system of dimension 2*n*.

The parallelized implementation of the Monte Carlo method uses space-parallel RK4 *m* times, once for each of the *m* sampled initial states. The implementation uses two levels of parallelization. The first level is a set of parallel threads over the samples used for simulations. Then, within each thread, another parallel set of threads are launched by space-parallel RK4. This is realized as one parallel job of $m \times n$ threads. Consequently, the Monte Carlo method has a complexity of $O(\frac{mn}{p})$. Since only the elementwise minima and maxima of the sampled states need to be stored, this method only requires as much memory as the growth bound method.

### Remark 1

A pseudocode of each parallel algorithm and a detailed discussion of their time and space complexities are provided in an extended version of this paper [29]. The extended version also contains additional details for the case studies that will be presented in the next section.

## Case Studies

In each of the case studies to follow, we report the time it takes PIRK to compute reachable sets for systems of varying dimension using all three of its methods on a variety of parallel computing platforms. We perform some of the same tests using the serial tool TIRA, to measure the speedup gained by PIRK’s ability to use massively parallel hardware.

We set a time limit of 1 h for all of the targeted case studies, and report the maximum dimensions that could be reached under this limit. The Monte Carlo method is given probabilistic parameters $\epsilon =\delta =0.05$ in each case study where it is used. We use four AWS machines for the computations with PIRK: m4.10xlarge which has a CPU with 40 cores, c5.24xlarge which has a CPU with 96 cores, g3.4xlarge which has a GPU with 2048 cores, and p3.2xlarge which has a GPU with 5120 cores. For the computations with TIRA, we used a machine with a 3.6 GHz Intel i7 CPU.

**Fig. 2.**
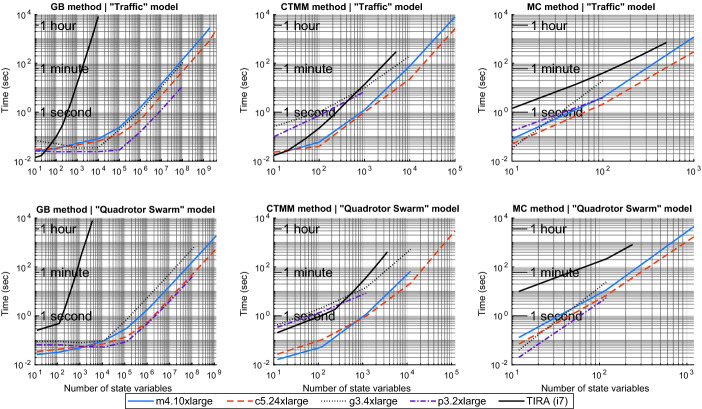
Logarithmic plots of the results for speed tests of the traffic model (first row) and the quadrotor swarm (second row). Speed test results for the serial interval reachability toolbox TIRA are also shown for the traffic model.

### *n*-link Road Traffic Model

We consider the road traffic analysis problem reported in [30], a proposed benchmark for formal controller synthesis. We are interested in the density of cars along a single one-way lane. The lane is divided into *n* segments, and the density of cars in each segment is a state variable. The continuous-time dynamics are derived from a spatially discretized version of the Cell Transmission Model [31]. This is a nonlinear system with sparse coupling between state variables.

The results of the speed test are shown in the first row of Figure 2. The machines m4.10xlarge and c5.24xlarge reach up to 2 billion and 4 billion dimensions, respectively, using the growth/contraction method, in 47.3 min and 44.7 min, respectively. Due to memory limitations of the GPUs, the machines g3.4xlarge and p3.2xlarge both reach up to 400 million in 106 s and 11 s, respectively.

The relative improvement of PIRK’s computation time over TIRA’s is significantly larger for the growth bound method than for the other two. This difference stems from how each tool computes the half-width of the reachable set from the radius dynamics. TIRA  solves the radius dynamics by computing the full matrix exponential using MATLAB’s expm, whereas PIRK directly integrates the dynamics using parallel Runge-Kutta. This caveat applies to Sect. 5.2 as well.

### Quadrotor Swarm

The second test system is a swarm of *K* identical quadrotors with nonlinear dynamics. The system dynamics of each quadrotor model are derived in a similar way to the model used in the ARCH-COMP 18 competition [32], with the added simplification of a small angle approximation in the angular dynamics and the neglect of Coriolis force terms. A derivation of both models is available in [33]. Similar to the n-link traffic model, this system is convenient for scaling: system consisting of one quadrotor can be expressed with 12 states, so the state dimension of the swarm system is $n=12K$. While this reachability problem could be decomposed into *K* separate reachability problems which can be solved separately, we solve the entire 12*K*-dimensional problem as a whole to demonstrate PIRK’s ability to make use of sparse interconnection.

The results of the speed test are shown in Fig. 2 (second row). The machines m4.10xlarge and c5.24xlarge reach up to 1.8 billion dimensions and 3.6 billion dimensions, respectively, (using the growth/contraction method) in 48 min and 32 min, respectively. The machines g3.4xlarge and p3.2xlarge both reach up to 120 million dimensions in 10.6 min and 46 s, respectively.

### Quadrotor Swarm with Artificial Potential Field

The third test system is a modification of the quadrotor swarm system which adds interactions between the quadrotors. In addition to the quadrotor dynamics described in Sect. 5.2, this model augments each quadrotor with an artificial potential field to guide it to the origin while avoiding collisions. This controller applies nonlinear force terms to the quadrotor dynamics that seek to minimize an *artificial potential* *U* that depends on the position of all of the quadrotors. Due to the interaction of the state variables in the force terms arising from the potential field, this system has a dense Jacobian. In particular, at least 25% of the Jacobian elements will be nonzero for any number of quadrotors.

**Table 1.** Results for running PIRK to compute the reach set of the quadrotors swarm with artificial potential field. “N/M” means that the machine did not have enough memory to compute the reachable set.

Method	No. of states	Memory (MB)	Time (seconds)			
			m4.10xlarge	c5.24xlarge	g3.4xlarge	p3.2xlarge
GB	1200	2.8	$\le$ 1.0	$\le$ 1.0	$\le$ 1.0	$\le$ 1.0
GB	12000	275.3	$\le$ 1.0	$\le$ 1.0	$\le$ 1.0	$\le$ 1.0
GB	120000	27,473.1	69.6	68.3	N/M	N/M
MC	1200	45.7	1.0	$\le$ 1.0	2.0	$\le$ 1.0
MC	12000	457.5	56.8	23.7	233.1	40.6
MC	120000	4577.6	$\ge$ 2h	3091.8	N/M	5081.0

Table 1 shows the times of running PIRK using this system on the four machines m4.10xlarge, c5.24xlarge, g3.4xlarge and p3.2xlarge in Amazon AWS. Due to the high density of this example, we focus on the memory-light growth bound and the Monte-Carlo methods. PIRK computed the reach sets of systems up to 120,000 state variables (i.e., 10,000 quadrotors). Up to 1,200 states, all machines solve the problems in less than one second. Some of the machines lack the required memory to solve the problems requiring large memory (e.g., 27.7 GB of memory is required to compute the reach set of the system with 120,000 state variables using the growth bound method).

### Heat Diffusion

The fourth test system is a model for the diffusion of heat in a 3-dimensional cube. The model is based on a benchmark used in [7] to test a method for numerical verification of affine systems. A model of the form $\dot{x} = f(t,x,p)$ which approximates the heat transfer through the cube according to the heat equation can be obtained by discretizing the cube into an $\ell \times \ell \times \ell$ grid, yielding a system with $\ell ^3$ states. The temperature at each grid point is taken as a state variable. Each spatial derivative is replaced with a finite-difference approximation. Since the heat equation is a linear PDE, the discretized system is linear.

We take a fixed state dimension of $n=10^9$ by fixing $\ell =1000$. Integration takes place over $[t_0,t_1]=[0,20]$ with time step size $h=0.02$. Using the Growth bound method, PIRK solves the problem on m4.10xlarge in 472 min, and in 350.2 min on c5.24xlarge. This is faster than the time reported in [7] (30 h) using the same machine.

### Overtaking Maneuver with a Single-Track Vehicle

The remaining case studies focus on models of practical importance with low state dimension. Although PIRK is designed to perform well on high-dimensional systems, it is also effective at quickly computing reachable sets for low dimensional systems, for applications that require many reachable sets. The first such case study is single-track vehicle model with seven states, presented in [34].

**Fig. 3.**
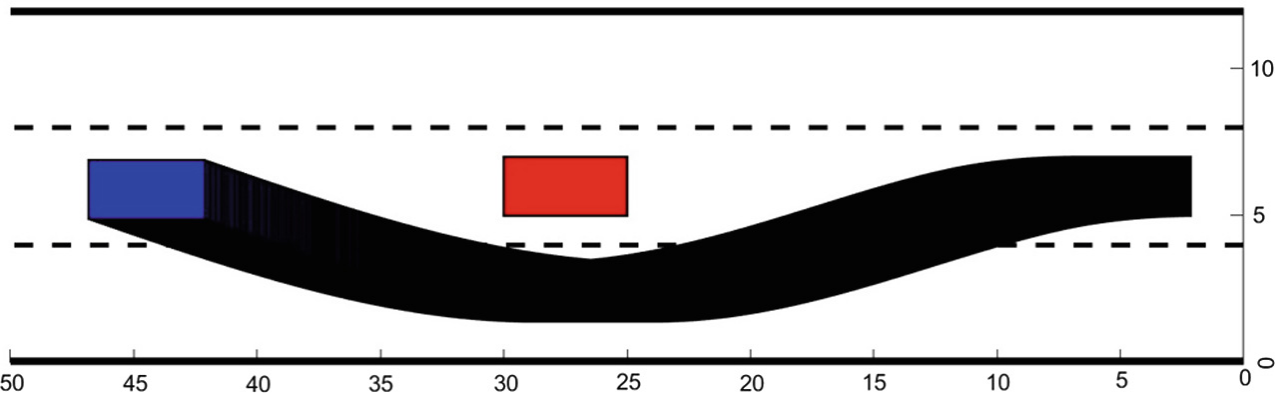
Reachable tube for the single-track vehicle.

We fix an input that performs a maneuver to overtake an obstacle in the middle lane of a 3-lane highway. To verify that the maneuver was safely completed, we compute reachable sets over a range of points and ensuring that the reachable set does not intersect any obstacles. We consider a step-size of 0.005 s in a time window between 0 and 6.5 s. We compute one reachable set at each time step, resulting in a “reachable tube” comprising 1300 reachable sets. PIRK computed the reachable tube in 0.25 s using the growth bound method on an i7 CPU (Fig. 3).

### Performance on ARCH Benchmarks

In order to compare PIRK’s performance to existing tools, we tested PIRK’s growth bound implementation on three systems from the ARCH-COMP’18 category report for systems with nonlinear dynamics [32]. This report contains benchmark data from several popular reachability analysis tools (C2E2, CORA, Flow$^*$, Isabelle, SpaceEx, and SymReach) on nonlinear reachability problems with state dimensions between 2 and 12.

**Table 2.** Results from running PIRK (growth bound method) to compute the reach sets for the examples reported in the ARCH-2018 competition.

Benchmark model	PIRK	CORA	CORA/SX	C2E2	Flow$^*$	Isabelle	SymReach
Van der Pol (2 states)	0.13	2.3	0.6	38.5	1.5	1.5	17.14
Laub-Loomis (7 states)	0.04	0.82	0.85	0.12	4.5	10	1.93
Quadrotor (12 states)	0.01	5.2	1.5	–	5.9	30	2.96

Table 2 compares the computation times for PIRK on the three systems to those reported by other tools in [32]. All times are in seconds. PIRK ran on an i9 CPU, while the others ran on i7 and i5: see [32] for more hardware details. PIRK solves each of the benchmark problems faster than the other tools. Both of the i7 and i9 processors used have 6 to 8 cores: the advantage of PIRK is its ability to utilize all available cores.

## Conclusion

Using a simple parallelization of interval reachability analysis techniques, PIRK is able to compute reachable sets for nonlinear systems faster and at higher dimensions than many existing tools. This performance increase comes from PIRK’s ability to use massively parallel hardware such as GPUs and CPU clusters, as well as the use of parallelizable simulation-based methods. Future work will focus on improving the memory-usage of the mixed monotonicity and Monte-Carlo based methods, including an investigation of adaptive sampling strategies, and on using PIRK as a helper tool to synthesize controllers for high-dimensional systems.

## Footnotes

1 PIRK is publicly available at https://github.com/mkhaled87/pFaces-PIRK.

2 While these non-idealities will be present in real systems and slow down computation, they should not affect the asymptotic complexity.
